# Traditional Removal Strategies Mitigate Shrub Encroachment Driven by Canopy Competition on the Tibetan Plateau

**DOI:** 10.1002/ece3.72682

**Published:** 2025-12-12

**Authors:** Jianping Yang, Peixi Su, Xianhong Meng, Zijuan Zhou, Rui Shi, Xinjing Ding

**Affiliations:** ^1^ State Key Laboratory of Cryospheric Science and Frozen Soil Engineering, Northwest Institute of Eco‐Environment and Resources Chinese Academy of Sciences Lanzhou China; ^2^ Zoige Plateau Wetland Ecosystem Research Station, Northwest Institute of Eco‐Environment and Resources Chinese Academy of Sciences Lanzhou China; ^3^ University of Chinese Academy of Sciences Beijing China; ^4^ College of Geographical Sciences Liaoning Normal University Dalian China

**Keywords:** Alpine meadow, elevation gradient, plant interactions, shrub encroachment, traditional ecological knowledge

## Abstract

Alpine grasslands of the Tibetan Plateau rank among Earth's ecologically critical yet vulnerable ecosystems. These ecosystems sustain pastoral livelihoods and preserve nomadic cultural traditions. However, climate warming combined with intensified anthropogenic pressures is accelerating shrub encroachment in these ecosystems. For centuries, Tibetan pastoralists have counteracted this process through targeted interventions: manual uprooting of dominant shrubs, controlled patch burning during dormant seasons, and rotational grazing systems coupled with strategic herbivore deployment to suppress woody seedlings. Despite their shrub management efficacy, the scientific rationale underlying Tibetan traditional ecological knowledge—the role of shrub‐herbaceous interaction variability in driving encroachment dynamics—remains understudied. We quantified shrub encroachment patterns across alpine meadows of the Zoige Plateau. Shrub encroachment dynamics were monitored across the elevation gradient (3400–3900 m), spanning the core elevational range of shrub encroachment. To identify shrub‐herbaceous interactions in driving encroachment dynamics, we conducted a manipulative experiment with four shrub treatments: shrub removal, canopy restriction, root exclusion, and ambient control. Our analysis identified that elevation imposed a strong linear constraint on short‐term encroachment rates (RSEI_Three_ = −0.06*E* + 0.28, *R* ^2^ = 0.83, *p* < 0.001; *E* (elevation, km)). No significant elevational effect was observed on long‐term encroachment rates (RSEI_Outset_; *p* > 0.05). Canopy competition (RII_Canopy_ = 0.06*E* − 0.41, *R* ^2^ = 0.81, *p* < 0.001) mainly explained 60.6% of the variation in encroachment dynamics, while root competition (RII_Root_ = −0.05*E* + 0.05, *R* ^2^ = 0.76, *p* < 0.001) accounted for 2.8%. Our findings highlight that canopy‐mediated suppression of herbaceous plants is the primary pathway driving encroachment, while root‐driven processes act as secondary mechanisms dependent on canopy architecture. Tibetan traditional methods directly disrupt key ecological drivers of shrub encroachment. By targeting shrub canopy removal, reducing herbaceous communities' light limitation, and weakening shrub competitive advantages that drive shrub encroachment, we promote the recovery of understory herbaceous communities.

## Graphical abstract

Rationale of Tibetan traditional ecological knowledge in mitigating shrub encroachment of alpine grasslands.

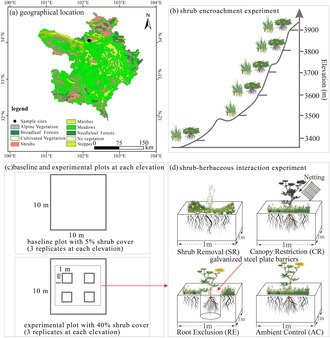

## 1 Introduction

Alpine grasslands, covering approximately 60% of the Tibetan Plateau (2.5 million km^2^), represent one of the most ecologically pivotal yet fragile ecosystems on Earth (Dong et al. 2020; Wang, Lv, et al. 2022). Situated at a mean elevation exceeding 4000 m, these grasslands are recognized as both a global biodiversity hotspot and a keystone component underpinning regional ecological security, pastoral livelihoods, and intangible cultural heritage (Dong 2023). Their unique position at the nexus of ecological sensitivity and anthropogenic pressures (e.g., overgrazing, land‐use change) highlights the imperative to disentangle their multifaceted roles in regulating biophysical processes and supporting coupled human‐natural systems (Xia et al. 2021).

Tibetan Plateau grasslands constitute a keystone component of global biogeochemical cycles, particularly carbon and water fluxes, while serving as a pivotal regulator of regional climate. As Asia's largest high‐altitude carbon sink, they store approximately 33.5 Gt of soil organic carbon in the 0–0.75 m profile (Wang et al. 2002). This sequestration capacity is driven by plant–soil feedbacks orchestrated by cold‐adapted sedges and grasses, particularly *Kobresia pygmaea* and *Stipa purpurea*, whose dense root mats mitigate soil erosion while enhancing carbon stabilization (Miehe et al. 2019). Beyond carbon sequestration, these grasslands are critical regulators of Asia's hydrological systems, feeding runoff in the Yangtze, Yellow, and Mekong headwaters through enhanced infiltration that buffers runoff variability and vegetation‐mediated insulation that slows permafrost thaw (Immerzeel et al. 2010; Wang et al. 2012). Economically, alpine grasslands underpin the Tibetan Plateau's pastoral economy, contributing over 40% of the region's agricultural GDP (Dong et al. 2020; Wu et al. 2023). Beyond direct pastoralism, these grasslands provide critical ecosystem services valued at $31.12 billion annually, including water filtration, waste treatment, and tourism values (Dong 2023).

Shrub encroachment—defined as the proliferation of native shrubs into historically herbaceous‐dominated grasslands and open woodlands—has emerged as a critical ecological regime shift across global drylands and alpine regions (O. W. Van Auken 2009; Eldridge et al. 2011; Naito and Cairns 2011). On the Tibetan Plateau (TP), the world's highest and most extensive plateau, this process is rapidly occurring, driven by synergistic interactions between rapid climate warming (0.16°C–0.67°C per decade since the 1950s; Kuang and Jiao 2016), anthropogenic activities (e.g., overgrazing; Geissler et al. 2019), and shifts in plant–soil feedback mechanisms (Zhao et al. 2025). Overgrazing—a legacy of intensified livestock production under China's grassland privatization policies—has degraded herbaceous competition, creating niche opportunities for shrub colonization (Harris 2010). From 2000 to 2015, the area of alpine grassland on the TP decreased by approximately 2000 km^2^, while shrublands expanded by 200 km^2^, indicating that 10% of the grassland loss was directly converted to shrub‐dominated systems (Fu et al. 2021). In the northeast TP, two shrubs— *Potentilla fruticosa* L. (syn. *Dasiphora fruticosa* (L.) Rydb., Zhang et al. 2024) and *Salix cupularis* Rehder—have rapidly encroached into alpine meadows, thereby inhibiting herbaceous plant growth and threatening the livelihoods of local pastoralists (Liu et al. 2023; Shi et al. 2023). Shrub encroachment degraded herbaceous communities via reduction of the water‐retentive soil mat layer. As documented by Liu et al. (2022), shrub‐root macropores increased soil infiltration by 100%–200%, driving a 10–24 g m^−2^ decline in herbaceous aboveground biomass and threatening alpine meadow biodiversity and ecosystem services. In addition, shrub encroachment also poses cascading risks to regional carbon sequestration, hydrological cycles, and pastoral livelihoods (Archer and Predick 2014). The threat of shrub encroachment is projected to intensify across the TP due to its accelerated warming rate and intensified land‐use pressures (Yao et al. 2019; Wang, Liao, et al. 2022); this motivates our hypothesis (H1) that increased elevation imposed a constraint on shrub encroachment.

Tibetan communities, with millennia‐old pastoral traditions, have developed traditional and local strategies to manage shrub encroachment, including: (1) Rotational grazing and targeted herbivory; seasonal livestock movements prevent localized overgrazing while exploiting sheep and yaks' dietary preferences for shrub seedlings. In Tianzhu Tibetan Autonomous County, herds are intentionally directed to shrub encroachment areas during early growth phases (May–June), reducing shrub biomass by 11.3%–83.5% compared to ungrazed controls (Wang, Li, et al. 2022); (2) Prescribed patch burning, according to Wangchuk et al. (2013), six consecutive years of pre‐snowmelt spring burning on senescent shrub patches reduces shrub cover by 60%–80% while preserving grass meristems. Additionally, post‐fire ash deposition also temporarily enriches soil phosphorus (Lambers et al. 2022), boosting grass recovery; (3) Manual shrub removal; household‐cooperative uprooting of juvenile shrubs (e.g., *Salix oritrepha* C.K.Schneid.) during autumn minimizes soil disturbance. The essence of Tibetan tradition and local management in addressing shrub encroachment is removing or weakening shrubs' competitive advantages. Plant–plant interactions are key factors in determining community structure, population dynamics, and distribution patterns (Brooker 2006; Anthelme et al. 2007). Shrub‐herbaceous interactions play a significant role in either facilitating or inhibiting shrub encroachment (Pierce et al. 2019; Marsman et al. 2021; Drees et al. 2023). For example, overgrazing is hypothesized to drive shrub encroachment by releasing woody plants from grass competition and reducing fire frequency in grasslands (O. W. Van Auken 2000). In shrub‐herbaceous interactions, aboveground competition for light is typically asymmetric, as taller (or larger) shrubs overshadow herbaceous plants. In contrast, belowground competition may exhibit greater symmetry as soil resources (e.g., water, mobile nutrients) are relatively homogeneous in distribution (Cahill Jr and Casper 2000; Hajek et al. 2015). This mechanistic framework led to our hypothesis (H2) that aboveground asymmetric competition for light exerts a stronger influence on shrub encroachment dynamics than symmetric belowground competition for soil nutrients and water. Although plant interaction‐mediated ecological regime shifts have been extensively quantified through Competitors, Stress‐tolerators, Ruderals (CSR) frameworks (Grime 1977), the role of shrub‐herbaceous interaction variability in driving encroachment dynamics remains poorly understood.

A mechanistic understanding of shrub encroachment and its drivers is critical to advance the restoration and management of degraded grasslands on the TP. From 2020 to 2023, we conducted two complementary experiments to dissect shrub encroachment drivers: (1) The Elevation Gradient Experiment assessed shrub encroachment dynamics across five altitudes (3400, 3600, 3700, 3800, and 3900 m a.s.l.) to quantify altitudinal effects; and (2) The Shrub Manipulation Experiment—shrub removal (SR), canopy restriction (CR), root exclusion (RE), and ambient control (AC)—in three independent replicate plots per elevation, measuring shrub‐herbaceous interactions via the Relative Interaction Index (RII). This integrated design specifically addressed: (i) elevation‐dependent modulation of shrub encroachment, and (ii) how shrub‐herbaceous interactions, specifically aboveground versus underground competition, affect shrub encroachment.

## 2 Materials and Methods

### 2.1 Study Site

**FIGURE 1:**
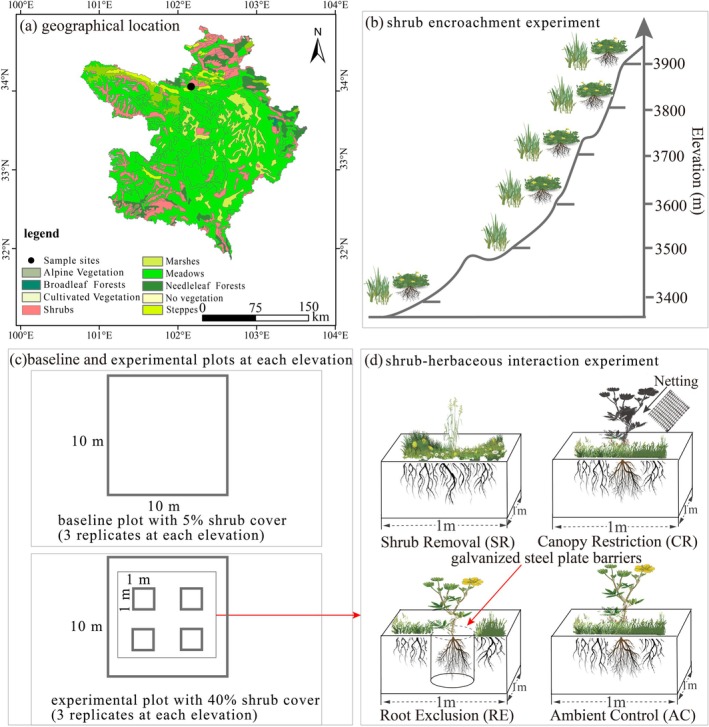
Schematic diagram of shrub encroachment and shrub‐herbaceous interaction experiments. (a) geographical location of sampling sites, (b) shrub encroachment experiment, (c) baseline and experimental plots at each elevation, and (d) Shrub‐herbaceous experiment; AC, ambient control; CR, canopy restriction; RE, root exclusion; SR, shrub removal.

The study was conducted in the alpine shrub meadow (sensu Yashiro et al. 2010) of the Zoige Plateau (33°10′ to 34°06′ N, 101°36′ to 103°25′ E) in the northeastern Tibetan Plateau (Figure 1a). Dominant soils are classified as silt clay loam (sand: silt: clay = 31.2:56.0:12.8) according to the USDA soil taxonomy. The region experiences a continental plateau semi‐humid climate (Bai et al. 2013). The seasons of rainfall and heat coincide. Diurnal air temperature variations are significant, with a mean daily range of 15.3°C (SD = 4.1°C) in summer. Separately, the site's absolute temperature extremes reach −33°C and 28°C based on Bai et al.'s (2013) 40‐year records (1961–2000). The plant growing season is from June to September. Across the study period (2020–2023), mean annual air temperatures varied from 0.6°C to 1.2°C; mean annual precipitation ranged from 600 to 800 mm. Frost seasons persisted 245 to 329 days annually. The annual potential evapotranspiration (PET) exceeds precipitation (Cheng et al. 2020; Yang et al. 2022).

The vegetation exhibited a mosaic structure dominated by *Dasiphora fruticose* shrubs (mean height: 0.6 m ± 0.15 SD; canopy cover: 40% ± 20% SD) competing with C_3_ graminoids (*Elymus nutans*, *Poa pratensis* ), sedges (*Carex parvula*, *Carex sargentiana*, *Carex alatauensis*), and *Potentilla anserina* , etc.

### 2.2 Experimental Design and Sampling Strategy

To investigate shrub encroachment dynamics along an elevation gradient (3400–3900 m) in the alpine shrub meadow at Gamaliang (34.07° N, 102.17° E), we established two types of permanent monitoring plots: baseline plots and experimental plots. Plots were positioned at 100‐m intervals across five target elevations (3400, 3600, 3700, 3800, and 3900 m; the 3500 m site was excluded due to the absence of *D. fruticosa* ; Figure 1b). Three independent replicate plots per type were established at each elevation, totaling 30 plots (2 types × 5 elevations × 3 replicates). All plots occupied uniform south‐facing slopes (10°–12° gradient), with topographic parameters quantified by RTK‐GPS (Trimble R12; ±1 cm vertical accuracy). Measurements indicated a mean slope of 11° ± 2° SD, an aspect of 190°–220° (south‐southwest), and planar surface curvature (mean profile curvature = 0.02 ± 0.03). (i) Baseline plots: At each elevation, three 10 × 10 m plots were maintained at a shrub cover of 5.0% through manual removal or transplantation of *D. fruticosa* (Figure 1c). Cover was quantified quarterly using the point‐intercept method (Elzinga and Salzer 1998). (ii) Experimental plots: At each elevation, three 10 × 10 m plots were maintained at a target shrub cover of 40% by adding or removing shrubs and ensuring that all shrubs exhibited comparable structural attributes (Figure 1c). Throughout the experiment, *D. fruticosa* was maintained as the sole woody species in all plots. The herbaceous community composition was standardized across plots by quarterly transplantation of target species and monthly removal of all non‐target species; the composition of herbaceous plant communities is shown in Table S2. This design encompasses the predominant elevation ranges where shrub encroachment manifests across the Zoige Plateau. The spatial sequence is nested within China's Ecological Conservation Redline Zone (established in 2015), thereby minimizing historical grazing impacts while preserving natural successional trajectories.

To investigate elevation‐dependent modulation of shrub‐herbaceous competition, within each experimental plot, four 1 × 1‐m quadrats were randomly assigned to four shrub treatments (*n* = 60 total) (Figure 1d):

1. Shrub Removal (SR): complete above‐ and below‐ground excision of *D. fruticosa* was implemented in June 2017. SR tested shrub‐herbaceous competition release.
2. Canopy Restriction (CR): shoot containment was achieved using UV‐stable polyethylene netting (1 mm × 1 mm mesh; 1‐mm^2^ opening area), reducing photosynthetically active radiation (PAR) by 90% for the shoot, as quantified with LI‐190R quantum sensors (LI‐COR Biosciences). CR was designed to isolate light competition by reducing PAR to shoots by 90%, while allowing below‐ground interactions between shrub and herbaceous plants.
3. Root Exclusion (RE): Annular trenches (5 cm width) were excavated around target shrubs, followed by the installation of perforated galvanized steel plate barriers (1.5 mm thickness, 80 cm depth, pore diameter: 0.15 mm) to allow soil water exchange and microbial transfer, and final backfilling with native soil. Barriers extended 2 cm aboveground to inhibit lateral root expansion through surface pathways. CR was designed to isolate below‐ground competition by root barriers, while allowing above‐ground interactions between shrubs and herbaceous plants.
4. Ambient Control (AC): Unmanipulated shrubs with 40% cover.

All manipulations were implemented in June 2017. Barrier integrity was verified through annual inspections until 2023.

During August surveys in 2020 and 2023, all plots (baseline and experimental) were assessed for species composition via the Braun‐Blanquet scale. Specifically, for *D. fruticosa* , we quantified relative cover (%), stem density (the number of stems per unit area, e.g., stems·m^−2^), and relative height using Greig‐Smith (1983) protocols. In each quadrat of experimental plots, aboveground biomass of herbaceous species was harvested from 1‐m^2^ quadrats. Samples were oven‐dried at 80°C until constant mass (typically 72 h), then weighed to obtain dry biomass using a precision balance (±0.01 g).

### 2.3 Shrub Encroachment Index

The Relative Shrub Encroachment Index (RSEI) integrates three demographic parameters to quantify *D. fruticosa* encroachment dynamics: density (reflecting recruitment success), canopy cover (indicating spatial dominance), and height (capturing light competition capacity)—collectively representing the ‘encroachment triad’ of woody plant encroachment (Lunt et al. 2010; Kulmatiski and Beard 2013). Unlike the steppe shrub encroachment index (SSEI) of Song and Wang (2022) (which integrates biodiversity [Margalef's R] with absolute structural values), RSEI quantifies the normalized difference in shrub structure between two time periods. This distinction reflects divergent research aims: SSEI evaluates ecosystem integrity, while our index targets encroachment dynamics. Zero‐centering allows bidirectional comparison (e.g., grassland to shrubland vs. shrubland to grassland transitions). The RSEI is calculated as:

$$\text{RSEI}=\frac{\left(\frac{D_{\mathrm{rel},\;e}-D_{\mathrm{rel},b}}{D_{\mathrm{rel},\;e}+D_{\mathrm{rel},\;b}}\right)+\left(\frac{C_{\mathrm{rel},e}-C_{\mathrm{rel},b}}{C_{\mathrm{rel},e}+C_{\mathrm{rel},b}}\right)+\left(\frac{H_{\mathrm{rel},e}-H_{\mathrm{rel},b}}{H_{\mathrm{rel},e}+H_{\mathrm{rel},b}}\right)}{3}$$

*D*_rel_: Relative density (stems/m^2^), *D*_rel,b_: *D*_rel_ at baseline (stems/m^2^), *D*_rel,e_: *D*_rel_ at endpoint (stems/m^2^); *C*_rel_: Relative canopy cover (%), *C*_rel,b_: *C*_rel_ at baseline (%), *C*_rel,e_: *C*_rel_ at endpoint (%); *H*_rel_: Relative height (cm), *H*_rel,b_: *H*_rel_ at baseline (cm), *H*_rel,e_: *H*_rel_ at endpoint (cm).

RSEI ranges between −1 and 1, with zero indicating shrub equilibrium (no changes in density, cover, and height); Shrub encroachment: RSEI > 0, the normalized difference sum of shrub relative density, shrub relative canopy, and shrub relative height is positive; Shrub retreat: RSEI < 0, the normalized difference sum of shrub relative density, shrub relative canopy, and shrub relative height is negative.

We developed two distinct RSEI metrics to assess encroachment dynamics, RSEI_Three_ characterizes shrub encroachment between 2020 and 2023, while RSEI_Outset_ evaluates shrub encroachment from reference meadow conditions (initial shrub onset) to 2023 endpoint states. A 5% shrub cover threshold was applied to differentiate meadows from shrublands, as per China's national standard *Technical Specifications for Assessment of Degradation, Sandification, and Salinization of Rangelands* (GB/T 19377‐2003).

### 2.4 Shrub‐Herbaceous Interaction Index

We quantified shrub‐herbaceous interactions using the Relative Interaction Index (RII; Armas et al. 2004), a metric ranging from −1 (complete competition) to 1 (complete facilitation). The RII was calculated as:

$$\mathrm{RII}=\frac{B_{w}-B_{o}}{B_{w}+B_{o}}$$

Here, *B*_*w*_ and *B*_*o*_ represent herbaceous biomass in the presence (whole plant, or only canopy, or only root) and absence of *D. fruticosa* shrubs, respectively. We calculated three distinct RII metrics to disentangle shrub effects:

$${\mathrm{RII}}_{\text{Shrub}}=\frac{B_{\mathrm{ac}}-B_{\mathrm{sr}}}{B_{\mathrm{ac}}+B_{\mathrm{sr}}}$$

where *B*_*ac*_ and *B*_*sr*_ are herbaceous biomass under Ambient Control (AC) and Shrub Removal (SR) treatments, respectively. The RII_Shrub_ represents whole‐shrub effect.

$${\mathrm{RII}}_{\text{Canopy}}=\frac{B_{\mathrm{re}}-B_{\mathrm{sr}}}{B_{\mathrm{re}}+B_{\mathrm{sr}}}$$

where *B*_*re*_ and *B*_*sr*_ denote herbaceous biomass under Root Exclusion (RE) and SR treatments, respectively. The RII_Canopy_ denotes shrub canopy effect on herbaceous communities.

$${\mathrm{RII}}_{\text{Root}}=\frac{B_{\mathrm{cr}}-B_{\mathrm{sr}}}{B_{\mathrm{cr}}+B_{\mathrm{sr}}}$$

where *B*_*cr*_ and *B*_*sr*_ are herbaceous biomass under Canopy Restriction (CR) and SR treatments, respectively. RII_Root_ quantifies shrub root effect on herbaceous communities.

To assess canopy‐root interactions, we computed RII_C×R_ as:

$${\mathrm{RII}}_{C\times R}={\mathrm{RII}}_{\text{Shrub}}-{\mathrm{RII}}_{\text{Canopy}}-{\mathrm{RII}}_{\text{Root}}$$

RII_C×R_ represents the specific effect of interactions between the shrub canopy and root systems, following the framework established by Cahill Jr (2002).

### 2.5 Statistical Analysis

Q‐Q plots and Bartlett's test were used to assess the normality and homogeneity of variance for biomass data, respectively. Data not fitting a normal distribution were log‐transformed to improve normality. Variation in relative interaction index (RII) and relative shrub encroachment index (RSEI) across the elevation gradient was assessed by one‐way ANOVA with Tukey's HSD post hoc tests. Linear regressions were used to quantify RII and RSEI dynamics across the elevation gradient. We screened collinearity among relative interaction indices (RIIs) using pairwise Pearson correlation coefficients and excluded variables with correlations ≥ 0.8 (e.g., RII_shrub_) to avoid multicollinearity. We conducted partial correlation analysis to determine whether and how the effect of a particular RII depended on other RIIs. We estimated the binary relationship between other RIIs and the RSEI by controlling one RII and using zero‐order correlation and partial correlation (Pearson correlation). We quantified the relative contributions of RIIs to RSEIs using variance partitioning analysis (VPA). This method was further applied to assess RIIs' effects on three shrub encroachment parameters: density, canopy cover, and height in turn.

All data analyses were conducted using R version 3.6.3. The homogeneity of variance was assessed using the car package (Fox et al. 2007), The partial correlation analysis was performed using the ppcor package (Kim 2015). Variation partitioning analysis was conducted using the vegan package (Oksanen et al. 2007).

## 3 Results

### 3.1 Shrub Encroachment Dynamics

**FIGURE 2:**
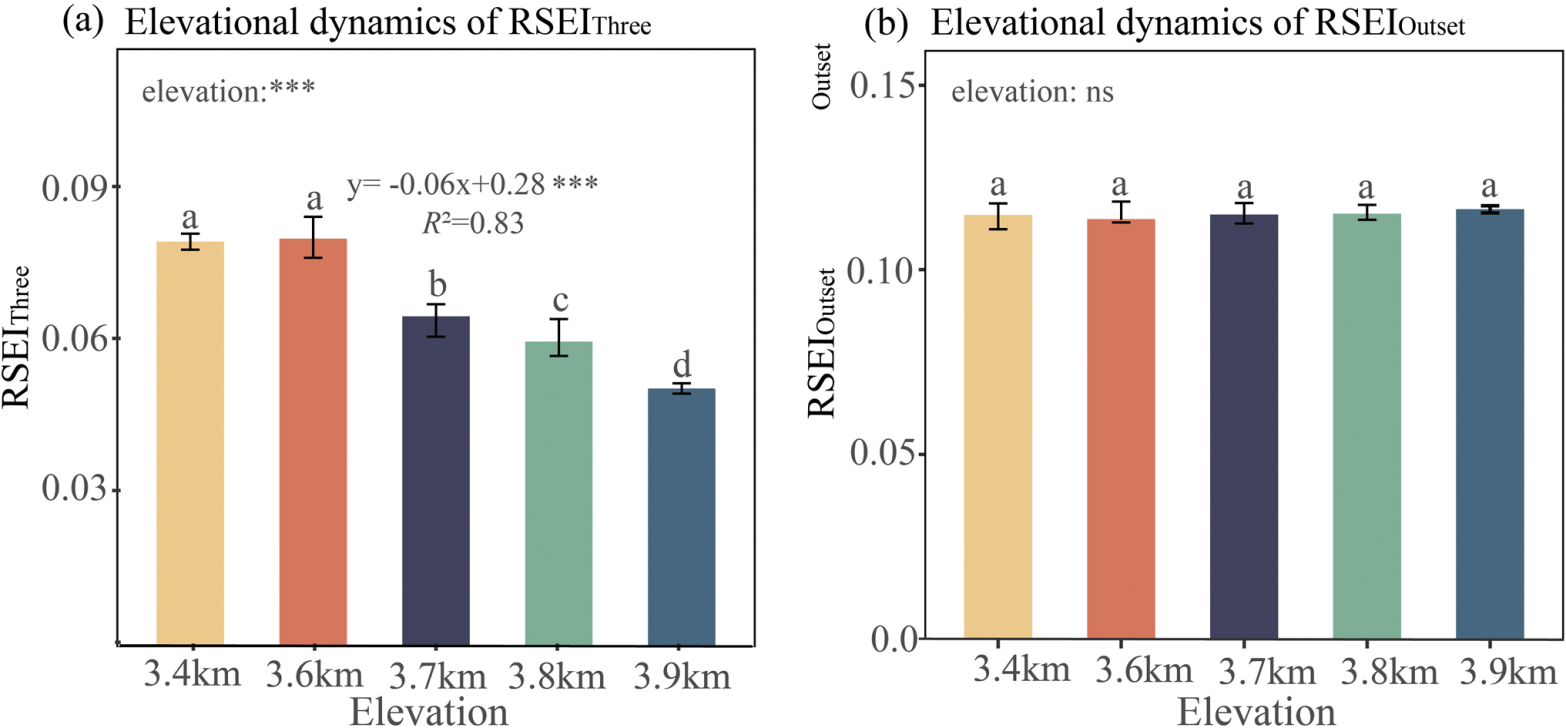
Elevational dynamics of shrub encroachment. (a) Elevational dynamics of RSEI_Three_, (b) Elevational dynamics of RSEI_Outset_; RSEI_Three_: Relative shrub encroachment index between 2020 and 2023, RSEI_Outset_: Relative shrub encroachment index between outset (5% shrub cover) and 2023. The RSEI_Three_ and RSEI_Outset_ values are dimensionless; significance levels for elevation and regression coefficients are denoted by asterisks: ***p < 0.001. The goodness‐of‐fit is evaluated by *R* ^*2*^; for multiple comparisons, different superscript letters indicate significant differences (Tukey's HSD test, *α* = 0.05), where shared letters imply non‐significant differences.

Between 2020 and 2023, divergent shrub encroachment trajectories occurred across the elevation gradient, with significant heterogeneity in RSEI_Three_ values among elevation zones (one‐way ANOVA, *p* < 0.001). RSEI_Three_ exhibited a significant 25% decline across the 500 m elevation gradient (3400 m: 0.079 vs. 3900 m: 0.050; Tukey's HSD *p* < 0.001). RSEI_Three_ exhibited a strong linear decline along the elevation gradient, decreasing by 6% per 1000‐m rise in elevation (RSEI_Three_ = −0.06*E* + 0.28, *R* ^2^ = 0.83, *p* < 0.001, *E* (elevation, km)) (Table S1; Figure 2a).

Elevation exhibited no significant influence on RSEI_Outset_ (*p >* 0.05), and pairwise comparisons (Tukey's HSD) across elevational bands revealed no significant differences (*p* > 0.05; Table S1; Figure 2b).

Shrub encroachment dynamics revealed phase‐specific decoupling in elevational responses between short‐term (RSEI_Three_) and long‐term encroachment (RSEI_Outset_) stages. The marginal rate of RSEI_Three_ remained constant across the elevation gradient (*β* = −0.06). In contrast, RSEI_Outset_ exhibited no significant elevational dependency.

### 3.2 Shrub‐Herbaceous Competition Variations

**FIGURE 3:**
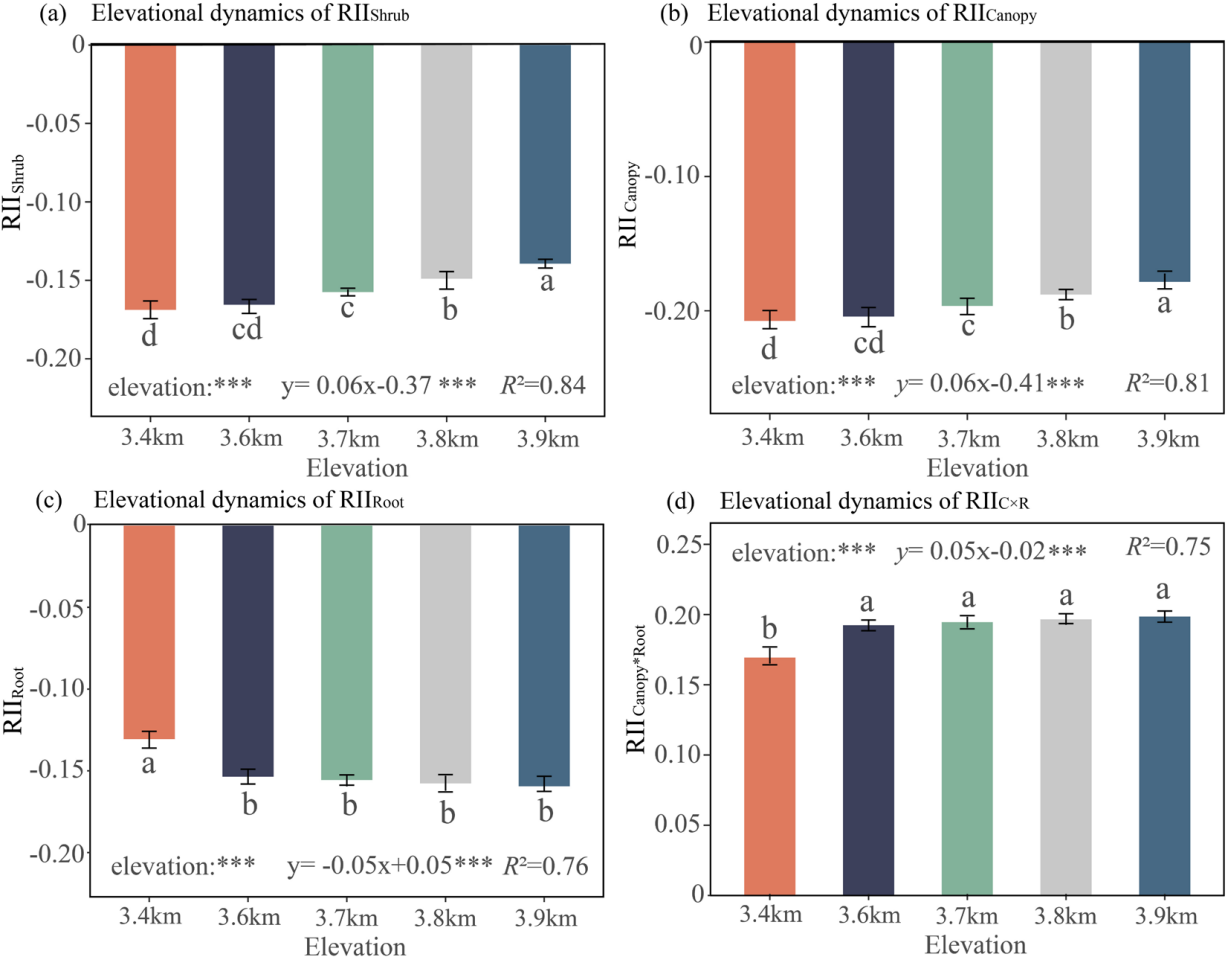
Elevational dynamics of relative interaction index. (a) Elevational dynamics of RII_Shrub_, (b) Elevational dynamics of RII_Canopy_, (c) Elevational dynamics of RII_Root_, and (d) Elevational dynamics of RII_C×R_; RII_Shrub_: Relative interaction index of shrub, RII_Canopy_: Relative interaction index of shrub canopy, RII_Root_: Relative interaction index of shrub root, RII_C×R_: Relative interaction index of shrub and root interaction. The RII_Shrub_, RII_Canopy_, RII_Root_, and RII_C×R_ values are dimensionless; significance levels for elevation and regression coefficients are denoted by asterisks: ***p < 0.001. The goodness‐of‐fit is evaluated by *R* ^2^; for multiple comparisons, different superscript letters indicate significant differences (Tukey's HSD test, *α* = 0.05), where shared letters imply non‐significant differences.

Shrub‐herbaceous interactions varied significantly along the elevation gradient, with pronounced heterogeneity in all interaction indices (RII_Shrub_, RII_Canopy_, RII_Root_, RII_C×R_) across elevation zones (one‐way ANOVA, *p* < 0.001). Linear regression analyses revealed strong along‐gradient patterns for all interaction indices, with *R* ^2^ values ranging from 0.75 to 0.98 (all *p* < 0.001; see Figure 3 for individual regression plots). Intact shrubs consistently competed with herbaceous species along the gradient (RII_Shrub_ = −0.17 to −0.14, Table S1; Figure 3a), driven by synergistic suppression from both canopy (RII_Canopy_ = −0.21 to −0.18, Table S1; Figure 3b) and root systems (RII_Root_ = −0.16 to −0.13, Table S1; Figure 3c). Notably, this competition was partially offset by antagonistic canopy‐root facilitation (RII_C×R_ = 0.17 to 0.20, Table S1; Figure 3d), which compensated for 53%–63% of total competitive intensity. Elevation modulated these interactions differentially. RII_Shrub_ (0.06E − 0.37, *R* ^2^ = 0.84, *p* < 0.001, Figure 3a), RII_Canopy_ (0.06E − 0.41, *R* ^2^ = 0.81, *p* < 0.001, Figure 3b), and RII_C×R_ (0.05E − 0.02, *R* ^2^ = 0.75, *p* < 0.001; Figure 3d) increased linearly with elevation, whereas RII_Root_ declined linearly (−0.05E + 0.05, *R* ^2^ = 0.76, *p* < 0.001; Figure 3c).

Shrub suppression (RII_Shrub_), canopy suppression (RII_Canopy_), and canopy‐root interaction facilitation (RII_C×R_) all exhibited fixed marginal effects with increasing elevation (*E*), as quantified by their regression coefficients: *β*_Shrub_ = 0.06; *β*_Canopy_ = 0.06; *β*_C×R_ = 0.05 (Figure 3a,b,d). RII_Root_ showed a fixed marginal effect (*β*_Root_ = −0.05, Figure 3c).

### 3.3 Increasing Elevation Inhibits Shrub Encroachment by Diminishing Shrub Competitive Superiority Over Herbaceous Plants

#### 3.3.1 Decoupling of Interaction Pathways

**FIGURE 4:**
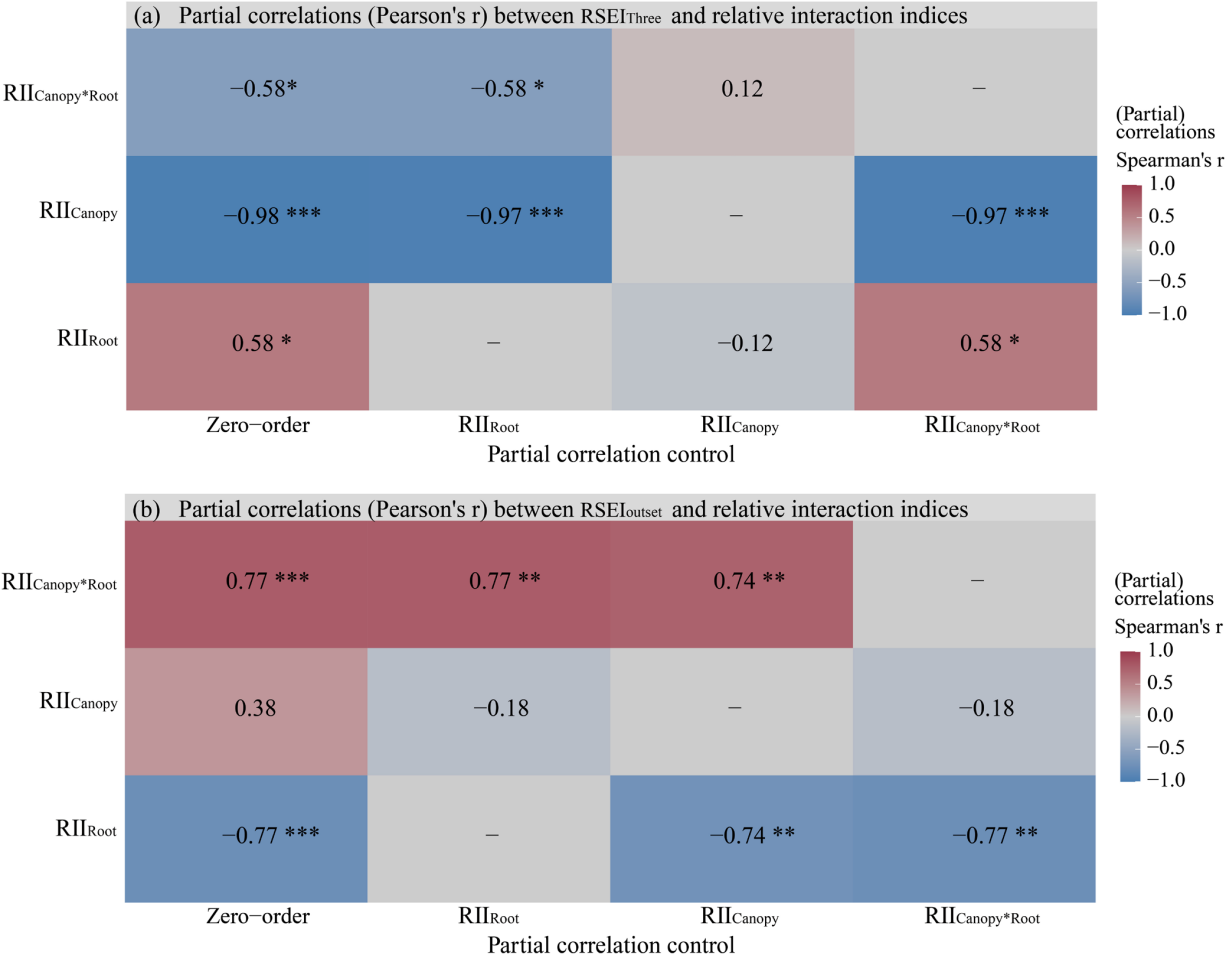
Partial correlations (Pearson's r) between relative shrub encroachment indices and relative interaction indices. (a) Partial correlations between RSEI_Three_ and relative interaction indices, (b) Partial correlations between RSEI_Outset_ and relative interaction indices; RSEI_Three_: Relative shrub encroachment index between 2020 and 2023, RSEI_Outset_: Relative shrub encroachment index between outset (5% shrub cover) and 2023, RII_Shrub_: Relative interaction index of shrub, RII_Canopy_: Relative interaction index of shrub canopy, RII_Root_: Relative interaction index of shrub root, RII_C×R_: Relative interaction index of shrub and root interaction. The RSEI_Three_ and RSEI_Outset_ values are dimensionless. The RII_Shrub_, RII_Canopy_, RII_Root_, and RII_C×R_ values also are dimensionless; color gradient intensity and numerical values denote the magnitude of correlation coefficients, and significance levels of the coefficients are denoted by asterisks (*p < 0.05, **p < 0.01, ***p < 0.001).

Partial correlation analysis demonstrated that RII_Canopy_ maintained strong negative correlations with RSEI_Three_ (Spearman's *r* = −0.99 to −0.97, *p* < 0.001) even after controlling RII_Root_ or RII_C×R_. In contrast, RII_Root_ and RII_C×R_ correlations collapsed from |*r*| ≈ 0.58 (*p* < 0.05) to |*r*| ≈ 0.12 (*p* > 0.05) when controlling for canopy effects (Figure 4a).

Partial correlation analysis further showed that RII_Root_ maintained strong negative correlations with RSEI_Outset_ (Spearman's *r* = −0.77, *p* < 0.001). In contrast, RII_C×R_ maintained strong positive correlations with RSEI_Outset_ (Spearman's *r* = 0.77, *p* < 0.001) (Figure 4b).

#### 3.3.2 Hierarchical Controls of Shrub‐Herbaceous Interactions

**FIGURE 5:**
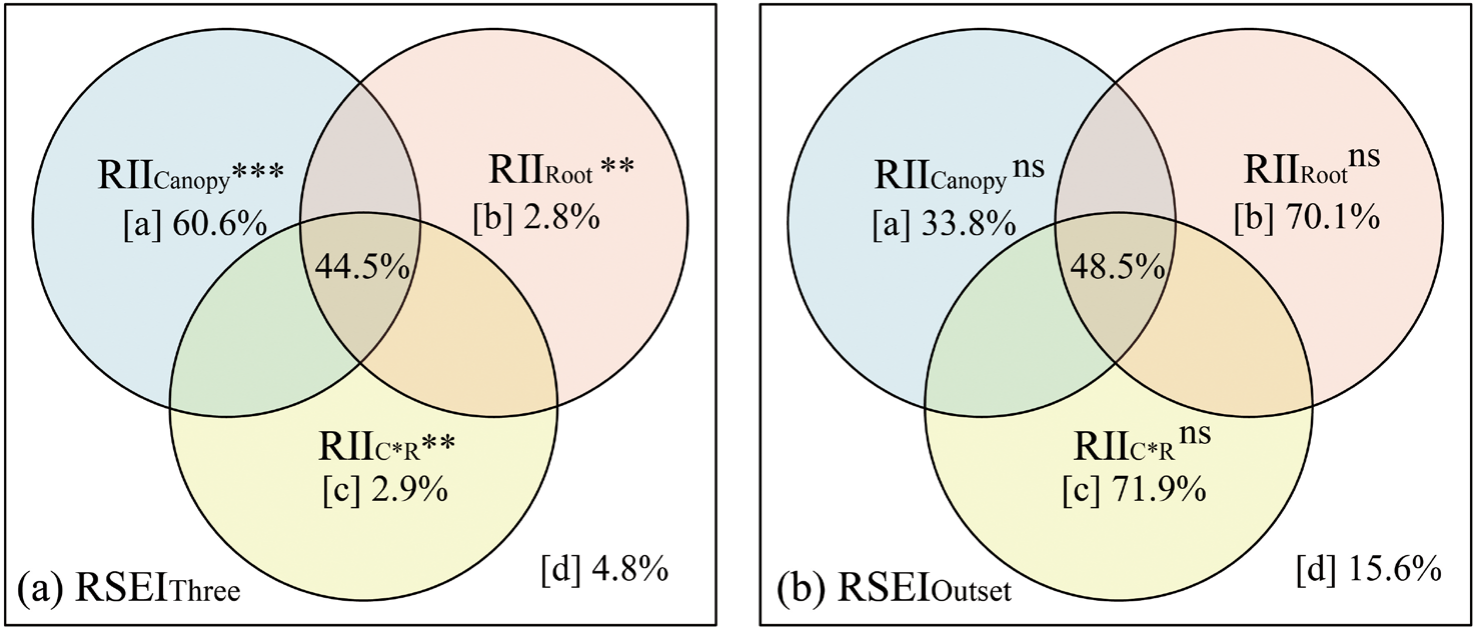
Variance partitioning analysis of relative shrub encroachment indices. (a) Variance partitioning analysis of RSEI_Three_, (b) Variance partitioning analysis of RSEI_Outset_; RSEI_Three_: Relative shrub encroachment index between 2020 and 2023, RSEI_Outset_: Relative shrub encroachment index between outset (5% shrub cover) and 2023, RII_Shrub_: Relative interaction index of shrub, RII_Canopy_: Relative interaction index of shrub canopy, RII_Root_: Relative interaction index of shrub root, RII_C×R_: Relative interaction index of shrub and root interaction. The RSEI_Three_ and RSEI_Outset_ values are dimensionless. The RII_Shrub_, RII_Canopy_, RII_Root_, and RII_C×R_ values also are dimensionless; [a] variation uniquely explained by RII_Canopy_, [b] variation uniquely explained by RII_Root_, [c]variation uniquely explained by RII_C×R_, and [d] unexplained variation (residuals). Overlapping areas between circles represent jointly explained variation by multiple predictors. Significance levels are denoted by asterisks (**p < 0.01, ***p < 0.001).

Variance partitioning analysis (VPA) attributed 95.2% of the variance in RSEI_Three_ to shrub‐herbaceous interactions. The VPA revealed a three‐tiered control: Shrub canopy competition (RII_Canopy_) was the dominant driver, accounting for 60.6% of RSEI_Three_ variation through suppression of herbaceous biomass (*p* < 0.001), while root competition (RII_Root_) and canopy‐root compensatory interactions (RII_C×R_) explained 2.8% and 2.9% RSEI variation, respectively (*p* < 0.01; Figure 5a). In contrast, shrub‐herbaceous interactions did not significantly explain the variance in RSEI_Outset_ (The *p* values for RII_Canopy_, RII_Root_, and RII_C×R_ were all > 0.05; Figure 5b).

#### 3.3.3 Pathways of Shrub Encroachment Regulation

**FIGURE 6:**
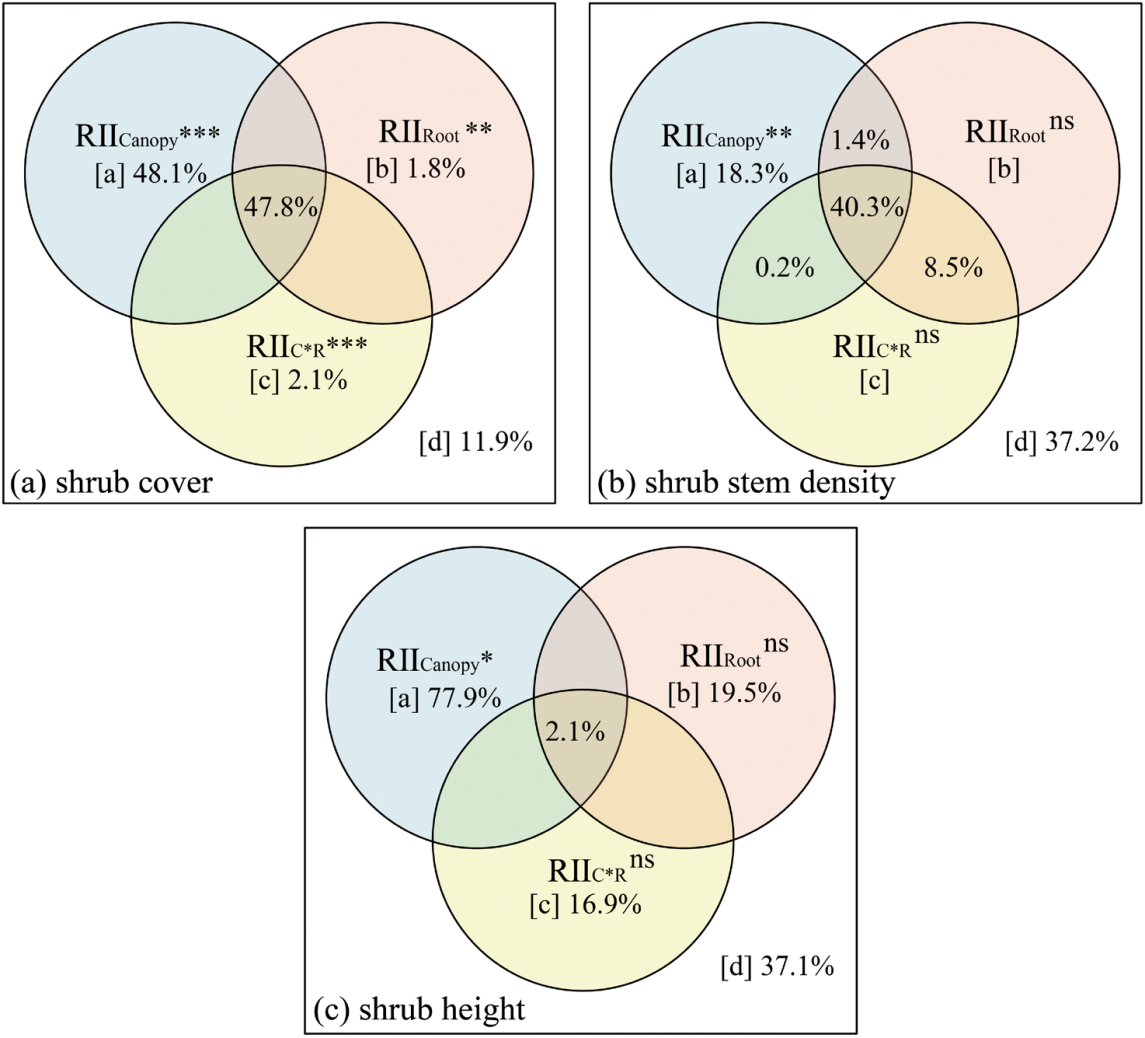
Variation partitioning analysis of shrub cover, stem density, and height. (a) Variance partitioning analysis of shrub cover, (b) Variance partitioning analysis of shrub stem density, (c) Variance partitioning analysis of shrub height; RII_Canopy_: Relative interaction index of shrub canopy, RII_Root_: Relative interaction index of shrub root, RII_C×R_: Relative interaction index of shrub and root interaction. The RII_Shrub_, RII_Canopy_, RII_Root_, and RII_C×R_ values also are dimensionless; [a] variation uniquely explained by RII_Canopy_, [b] variation uniquely explained by RII_Root_, [c] variation uniquely explained by RII_C×R_, and [d] unexplained variation (residuals). Overlapping areas between circles represent jointly explained variation by multiple predictors. Significance levels are denoted by asterisks (*p < 0.05, **p < 0.01, ***p < 0.001).

RII_Canopy_ exerted significant control over shrub encroachment dynamics, driving substantial increases in key structural parameters: shrub canopy cover rose by 48.1% (*p* < 0.001), stem density increased by 18.3% (*p* < 0.01), and shrub height expanded by 77.9% (*p* < 0.05) (Figure 6a). While RII_Root_'s direct facilitation of encroachment was quantitatively weaker than canopy‐mediated effects (path coefficients = 1.8%, Figure 6a), it significantly amplified shrub canopy cover (*p* < 0.01). In contrast, its effects on stem density (*p* > 0.05, Figure 6b) and shrub height (*p* > 0.05, Figure 6c) were non‐significant. RII_C×R_ significantly suppressed shrub encroachment dynamics, primarily through reducing canopy cover by 2.1% (*p* < 0.001, Figure 6a). No significant suppression was detected for stem density (*p* > 0.05, Figure 6b) or shrub height (*p* > 0.05, Figure 6c).

## 4 Discussion

### 4.1 Elevational Constraints on Shrub Encroachment Dynamics

Our findings revealed a critical decoupling between short‐term (RSEI_Three_) and long‐term shrub encroachment (RSEI_Outset_) along elevation gradients. The 25% decline in RSEI_Three_ across the 500‐m elevational gradient (3400–3900 m; *β* = −0.06/km, *p* < 0.001) reflects temperature‐mediated environmental filtering. This process suppresses shrub establishment under high‐elevation conditions, thereby constraining short‐term shrub encroachment in these zones. This pattern mirrored global observations of alpine treelines controlled by thermal thresholds (Körner 2021a), but extended this framework to shrub‐grass ecotones. Notably, the stability of RSEI_Outset_ across the elevation gradient (*p* > 0.05) suggested that shrub canopy closure triggers self‐reinforcing feedbacks through light monopolization (e.g., suppression of herbaceous plants), thereby progressively attenuating abiotic constraints associated with elevation (Clark and Bullock 2007; Boscutti et al. 2018). These biotic feedbacks progressively override abiotic constraints associated with elevation during long‐term shrub encroachment. Consequently, our results challenge the traditional paradigm of elevation as a static ecological constraint (Dullinger et al. 2004). Our study highlights phase‐dependent controls: abiotic associated with elevation filters dominated shrub recruitment (RSEI_Three_), while biotic competition governed long‐term shrub encroachment (RSEI_Outset_). This phase‐specific decoupling parallels recent reports of “abiotic‐biotic asymmetry” in shrub encroachment (Weber‐Grullon et al. 2022), where abiotic stressors had a significant effect but competition between grasses and shrub seedlings had no effect on the germination or survival of *Prosopis glandulosa* during recruitment stages.

The stability of RSEI_Outset_ across the elevation gradient implies that shrubs may employ adaptive plasticity to sustain long‐term dominance—likely through strong competitive ability for soil nutrients (Ram and Chawla 2024) or allelochemical suppression of herbaceous competitors (Valladares et al. 2016). Climate warming may disproportionately accelerate shrub encroachment at higher elevations by relaxing establishment barriers. This underscores the importance of distinguishing transient recruitment bottlenecks from persistent competitive hierarchies in predicting landscape‐scale encroachment trajectories under climate warming.

### 4.2 Elevational Modulation of Shrub‐Herbaceous Interactions

Alpine plants are often subjected to intense abiotic stress, which typically leads to facilitative interactions among plants being more commonly observed in these high‐elevation environments (Cavieres and Badano 2009; Ballantyne and Pickering 2015). However, in this study, shrubs consistently competed with herbaceous plants across all elevation gradients (RII_Shrub_ < 0), aligning with evidence that facilitation rarely dominates community‐level interactions along elevation gradients (Liancourt et al. 2017). This pattern likely arises because shrubs impose strong asymmetric competition on subordinate herbaceous species, generating net competitive outcomes at the community scale (Ram and Chawla 2024). The absence of consumer pressure (e.g., herbivory) in our system may further explain deviations from predictions of the Stress Gradient Hypothesis (SGH; Smit et al. 2009). Most empirical support for SGH derives from studies of one or a few species pairs, where stress‐tolerant beneficiaries interact with competitive benefactors under abiotic stress gradients dominated by non‐resource factors (Maestre et al. 2009; Michalet et al. 2014). In contrast, our study examined herbaceous communities—typically considered competitors rather than stress‐tolerant beneficiaries—thus appearing inconsistent with SGH predictions.

The elevation‐driven shifts in shrub‐herbaceous interactions (*R* ^2^ = 0.75–0.98) demonstrate that competition‐facilitations are dynamically recalibrated along environmental gradients. Synergistic suppression by shrub canopy and root systems drives encroachment through two mutually reinforcing mechanisms: (1) Canopy expansion facilitates root growth by enhancing photosynthate production (Brooker 2006); (2) Root system development facilitates canopy growth through niche preemption and resource acquisition (e.g., soil moisture and nutrients; Brooker 2006). These processes establish a positive feedback loop that amplifies shrub dominance. The negative RII values (RII_Canopy_ = −0.21 to −0.18, RII_Root_ = −0.16 to −0.13) quantify competitive suppression, while their synergistic interaction (i.e., non‐additive effects) drives the feedback loop. This aligns with Jardim et al. (2025) demonstrating combined above‐ and belowground competition as key encroachment drivers. However, the compensatory canopy‐root facilitation (RII_C×R_ = 0.17 to 0.20) unveils a novel interaction axis, where shrubs' nursing effects (e.g., hydraulic lifting) may partially facilitate herbaceous survival and growth under harsh conditions (Brooker et al. 2008).

The positive values in marginal interaction effects (*β*_Canopy_ = +0.06 vs. *β*_C×R_ = +0.05/km) align with the stress‐gradient hypothesis (Bertness and Callaway 1994; Ballantyne and Pickering 2015), where facilitative interactions intensify with increasing environmental stress. The significantly negative marginal effect of elevation on root competition intensity (*β*_Root_ = −0.05/km; *p* < 0.01) indicates increased belowground competition at higher elevations. We hypothesize that this increase may arise from limited nutrient availability in high‐elevation soils, where biological weathering is suppressed by low temperatures (Ram and Chawla 2024). Consequently, shrubs and herbs may compete more intensely due to diminished resource pools. Importantly, the 53%–63% competition offset by RII_C×R_ suggests that traditional single‐mechanism competition indices (e.g., RII_Shrub_ alone) may underestimate interaction complexity in vertically stratified ecosystems.

### 4.3 Elevation Modulates Shrub Encroachment by Altering Shrub‐Herbaceous Competition

Plant–plant interactions are key drivers of community structure, population dynamics, and species distribution patterns (Brooker 2006; Anthelme et al. 2007). These interactions may critically regulate shrub encroachment dynamics by mediating competitive hierarchies (Drees et al. 2023). In this study, *D. fruticosa* is a competitively superior species; shrub‐herbaceous interactions explained approximately 95% of the variance in RSEI_Three_, suggesting that shrub‐herbaceous interactions significantly influence shrub encroachment. This dominant role of biotic interactions aligns with prior evidence that shrub encroachment is driven by shrubs' competitive superiority in resource acquisition (Shi et al. 2023).

The hierarchical controls of elevation on shrub‐herbaceous interactions reveal a dynamic interplay between abiotic constraints and biotic competition in driving encroachment patterns. The partial correlation analyses unveil a striking temporal decoupling in shrub‐herbaceous interaction pathways. During short‐term encroachment (RSEI_Three_), canopy competition (RII_Canopy_) exhibited robust negative correlations with shrub encroachment (Spearman's *r* = −0.99 to −0.97) even when controlling for root effects, indicating shrubs' competitive superiority in light interception is the primary driver of early‐stage encroachment. The dominance of RII_Canopy_ in explaining 60.6% of RSEI_Three_ variation aligns with the well‐documented asymmetric nature of light competition, where taller shrubs exert disproportionate shading effects on herbaceous plants (Hajek et al. 2015). Furthermore, shrubs' perennial growth strategy enables sustained biomass accumulation and vertical expansion (Köchy and Wilson 2000), driving light preemption dominance in meadows characterized by adequate soil resources but intense shading regimes (Hautier et al. 2009). In this study, RII_Root_ explained 2.8% of RSEI_Three_ variation, but the collapse of RII_Root_ and RSEI_Three_ correlations when controlling for canopy effects (|*r*| ≈ 0.12) demonstrates that belowground interactions are hierarchically dependent on prior canopy dominance. The near‐complete explanation of RSEI_Three_ variance by shrub‐herbaceous interactions (95.2%) contrasts sharply with the non‐significant effects of these interactions on RSEI_Outset_. This discrepancy may arise from two non‐mutually exclusive mechanisms: (1) short‐term shrub‐herbaceous interactions fail to predict long‐term encroachment dynamics due to temporal decoupling of competition‐facilitation balances, or (2) abiotic filters (e.g., soil properties, dispersal limitation) override biotic controls in governing shrub persistence over extended timescales—a pattern congruent with alpine treeline dynamics where abiotic constraints dominate long‐term vegetation boundaries (Körner 2021b).

Previous studies have demonstrated that shrub encroachment rates exhibit cover‐dependent dynamics (Roques et al. 2001), a pattern consistent with our findings that superior light competition facilitated shrub encroachment through increases in relative canopy cover (+48.1%), stem density (+18.3%), and height (+77.9%).

### 4.4 Rationale and Efficacy of Tibetan Traditional Ecological Knowledge in Mitigating Shrub Encroachment

Traditional Ecological Knowledge (TEK) provides critical insights for mitigating alpine grassland degradation. Tibetan herders have historically employed targeted shrub removal practices—such as manual shrub removal, prescribed patch burning, and rotational grazing and targeted herbivory—to sustain pasture productivity (Wangchuk et al. 2013; Wang, Li, et al. 2022). These methods, adaptively refined over centuries, directly disrupt key ecological drivers of shrub encroachment. By targeting shrub canopy removal, reducing herbaceous communities' light limitation, and weakening shrub competitive advantages that drive shrub encroachment, thereby promoting the recovery of understory herbaceous communities.

Tibetan manual shrub removal directly disrupts shrub canopy architecture, reducing light interception capacity by 60%–80% within the first growing season post‐treatment. These practices shift competitive dynamics in favor of light‐demanding grasses by severing the positive feedback loop between canopy closure and understory light limitation. This aligns with evidence that canopy reduction facilitates understory productivity by enhancing access to photosynthetically active radiation (PAR; Feltrin et al. 2016). Controlled grazing further amplifies this effect: by preferentially browsing shrub seedlings, livestock curb stem recruitment and impede closed‐canopy formation. These practices are functionally analogous to the ecological role of large mammals in suppressing woody plant dominance (Stevens et al. 2016), demonstrating TEK's convergence with historical trophic regulation mechanisms.

While Tibetan methods primarily target aboveground structures, they indirectly alter belowground dynamics. Post‐fire ash deposition elevates soil pH and phosphorus availability, shifting competitive advantages to herbaceous species with rapid nutrient uptake and rhizome‐based clonal traits (Wangchuk et al. 2013). This contrasts with the natural state, where intact shrubs suppress herbaceous communities through synergistic aboveground light interception and belowground nutrient preemption. Critically, TEK avoids the pitfalls of mechanical removal (e.g., soil disturbance promoting shrub resprouting, Clarke et al. 2010) by leveraging phenological precision—burning in late winter when shrub carbohydrate reserves are depleted, thereby minimizing regenerative capacity.

Despite TEK's efficacy, climate warming threatens to undermine its long‐term sustainability. On the Tibetan Plateau, rising temperatures have prolonged the shrub growing season (Geissler et al. 2019), enhancing shrubs' capacity to regenerate after removal. Concurrently, herbaceous species—the intended beneficiaries of TEK—face intensified drought stress, which diminishes their competitive resilience (Mount et al. 2024). Collectively, these shifts risk decoupling the traditional “removal‐recovery” feedback cycle, necessitating hybrid adaptive strategies. Potential solutions include: (1) integrating TEK with assisted seeding of drought‐tolerant *Kobresia cultivars* to enhance grassland resistance, and (2) extending fallow periods between rotational grazing cycles to alleviate soil compaction, thereby improving herbaceous root development and countering shrub root competition.

The Stress Gradient Hypothesis (SGH) predicts a shift from competition to facilitation under increasing abiotic stress, yet our findings demonstrate persistent net shrub‐dominated competition across the elevational gradient. This discrepancy stems from SGH's original focus on non‐resource stress gradients (e.g., salinity, extreme temperatures) (Michalet et al. 2014), whereas alpine shrub encroachment is primarily driven by resource competition (light interception, soil moisture depletion). TEK's effectiveness arises from its empirical understanding of these ecological mechanisms: by targeting shrub resource monopolization strategies (e.g., reducing canopy cover density), traditional practices directly disrupt the resource‐driven encroachment process rather than mitigate secondary stress effects.

However, while our study assessed species interactions, it did not evaluate environmental factors beyond elevation (e.g., soil physicochemical properties); thus, their potential impacts on shrub encroachment remain unquantified. Moreover, the three‐year observation period is insufficient to capture slow demographic processes in alpine shrub communities. Key dynamics—such as sustained shrub recruitment and lag effects in belowground competition—may require longer timescales to manifest. Future research extending over decades is needed to fully evaluate how environmental variables and species interactions drive shrub encroachment.

## 5 Conclusions

Our analysis reveals that elevation gradient significantly suppresses short‐term shrub encroachment (*p* < 0.05), yet exhibits no statistically detectable effect on long‐term encroachment dynamics. The inhibitory effect of shrubs on herbaceous communities, quantified by the Relative Interaction Index for shrubs (RII_Shrub_), declines with elevation. Conversely, interaction effects between shrub canopy and root—measured through the Canopy‐Root Interaction Index (RII_C×R_)—intensify at higher elevation. Canopy competition (RII_Canopy_ = 0.06*E* − 0.41, *R* ^2^ = 0.81, *p* < 0.001) mainly explained 60.6% of the variation in encroachment dynamics, while root competition (RII_Root_ = −0.05*E* + 0.05, *R* ^2^ = 0.76, *p* < 0.001) accounted for 2.8%. These findings demonstrate that canopy‐mediated suppression of herbaceous plants constitutes the primary pathway driving shrub encroachment, while root‐driven processes operate as secondary mechanisms contingent on canopy structural complexity. Traditional Tibetan management practices effectively disrupt these ecological drivers. By targeting shrub canopy removal, reducing light limitation for herbaceous communities, and weakening shrub competitive advantages in resource acquisition, these methods promote the recovery of understory herbaceous diversity.

## Author Contributions

**Jianping Yang:** conceptualization (lead), data curation (lead), methodology (lead), software (equal), validation (equal), visualization (equal), writing – original draft (equal), writing – review and editing (equal). **Peixi Su:** conceptualization (equal), data curation (equal), investigation (equal), methodology (equal), validation (equal), visualization (equal), writing – review and editing (equal). **Xianhong Meng:** conceptualization (lead), data curation (equal), investigation (equal), methodology (lead), project administration (lead), software (equal), validation (equal), visualization (equal), writing – original draft (equal), writing – review and editing (equal). **Zijuan Zhou:** investigation (equal), methodology (equal), validation (equal), visualization (equal), writing – review and editing (equal). **Rui Shi:** investigation (equal), methodology (equal), visualization (equal), writing – review and editing (equal). **Xinjing Ding:** investigation (equal), methodology (equal), visualization (equal), writing – original draft (equal), writing – review and editing (equal).

## Funding

This work was supported by the National Science Fund for Distinguished Young Scholars of China, 42325502; the Foundation of the Science and Technology Research Plan of Gansu Province, 23JRRA654; the West Light Foundation for Western Cross Team of the Chinese Academy of Sciences, xbzg‐zdsys‐202215; the Lanzhou Youth Science and Technology Talent Innovation Project, 2024‐QN‐48; the Science and Technology Program of Gansu Province, 23JRRA608; the Leading Talent Support Program of Gansu Province.

## Conflicts of Interest

The authors declare no conflicts of interest.

## Supporting information


**Table S1:** Shrub encroachment indices, shrub‐herbaceous interaction indices, and results of analysis of variance for the difference across cover (one‐way ANOVAs).
**Table S2:** Standardized herbaceous community composition at each elevation (3400, 3600, 3700, 3800, and 3900 m).

## Data Availability

Data and R code for partial correlation analysis are available via figshare (https://figshare.com/articles/journal_contribution/Data_and_R_code_zip/30691958).
