# Supplementary material for: Traditional Removal Strategies Mitigate Shrub Encroachment Driven by Canopy Competition on the Tibetan Plateau
Source: Ecol Evol. 2025 Dec 12;15(12):e72682. doi: 10.1002/ece3.72682 (PMC12699206; doi:10.1002/ece3.72682)
Supplement: Supplementary file 1 — Table S1: Shrub encroachment indices, shrub‐herbaceous interaction indices, and results of analysis of variance for the difference across cover (one‐way ANOVAs). Table S2: Standardized herbaceous community composition at each elevation (3400, 3600, 3700, 3800, and 3900 m). [file ECE3-15-e72682-s001.docx]

***Ecology and Evolution***

**Article title: Traditional removal strategies mitigate shrub encroachment driven by canopy competition on the Tibetan Plateau**

**Table S1** Shrub encroachment indices, shrub-herbaceous interaction indices, and results of analysis of variance for the difference across cover (one-way ANOVAs)

Shrub encroachment indices

| Parameters | RSEI_Three_ | RSEI_Outset_ |
| --- | --- | --- |
| Units | No units | No units |
| 3400 m | 0.079±0.002a | 0.115±0.004a |
| 3600 m | 0.079±0.004a | 0.114±0.003a |
| 3700 m | 0.064±0.003b | 0.115±0.003a |
| 3800 m | 0.0593±0.003c | 0.115±0.002a |
| 3900 m | 0.05±0.001d | 0.121±0.001a |

RSEI_Three_: relative shrub encroachment index between 2020 and 2023, RSEI_Outset_: relative shrub encroachment index between outset (5% shrub cover) and 2023

Shrub-herbaceous interactions

| Parameters | RII_Shrub_ | RII_Canopy_ | RII_Root_ | RII_C*R_ |
| --- | --- | --- | --- | --- |
| Units | No units | No units | No units | No units |
| 3400 m | -0.169±0.006d | -0.208±0.007d | -0.131±0.005a | 0.169±0.003b |
| 3600 m | -0.166±0.005cd | -0.204±0.008cd | -0.154±0.005b | 0.192±0.002a |
| 3700 m | -0.157±0.003c | -0.196±0.007c | -0.156±0.003b | 0.195±0.002a |
| 3800 m | -0.149±0.006b | -0.188±0.004b | -0.158±0.005b | 0.197±0.001a |
| 3900 m | -0.139±0.003a | -0.178±0.006a | -0.159±0.004b | 0.199±0.001a |

RII_Shrub_: relative interaction index of shrub, RII_Canopy_: relative interaction index of shrub canopy, RII_Root_: relative interaction index of shrub root, RII_C×R_: relative interaction index of shrub and root interaction

**Table S2** Standardized herbaceous community composition at each elevation (3400, 3600, 3700, 3800, and 3900 m)

| **Species** | **Cover (%)** |
| --- | --- |
| *Elymus nutans* | 15 |
| *Poa pratensis* | 10 |
| *Carex parvula* | 5 |
| *Carex sargentiana* | 3 |
| *Carex alatauensis* | 2 |
| *Potentilla anserina* | 5 |
